# Formation of Nanoscale Al_2_O_3_ Protective Layer by Preheating Treatment for Improving Corrosion Resistance of Dilute Fe-Al Alloys

**DOI:** 10.3390/ma15227978

**Published:** 2022-11-11

**Authors:** Chenglong Li, Katharina Freiberg, Yuntong Tang, Stephanie Lippmann, Yongfu Zhu

**Affiliations:** 1 Key Laboratory of Automobile Materials, Ministry of Education, School of Materials Science and Engineering, Jilin University, Changchun 130022, China; 2 Otto Schott Institute of Materials Research, Friedrich Schiller University, 07743 Jena, Germany

**Keywords:** corrosion resistance, oxidation, heat treatment, Fe-Al alloy, ferroalloy

## Abstract

In this work, an attempt was made to improve the corrosion resistance of dilute Fe-Al alloys (1.0 mass% Al) by preheating treatment at 1073 K in H_2_ atmosphere. In comparison with pure Fe and unpreheated Fe-Al alloys, the resistance to oxidation at 673 K in pure O_2_ and to electrochemical corrosion in 5 wt.% NaCl solution is significantly improved for preheated Fe-Al alloys. This improvement is attributed to the formation of a 20 nm thin, but dense Al_2_O_3_ protective layer on the surface of preheated Fe-Al alloys.

## 1. Introduction

As one of the most widely used structural materials, ferroalloys are widely utilized in vehicle engineering, the petrochemical industry, machinery manufacturing, aerospace and marine engineering [1]. In modern society, high-temperature oxidation in air and electrochemical corrosion in wet air or chemical solution are the most common ways to cause ferroalloy failure, leading to economic losses and potential security risks [2]. Consequently, the high-temperature oxidation resistance [3,4] and corrosion resistance [5,6,7,8] of ferroalloys must be considered.

For ferroalloys, the commonly used anticorrosion methods include alloying, surface coating and electroplating. Although all mentioned methods improve the oxidation resistance and the electrochemical corrosion resistance of ferroalloys, they require additional process steps that are costly, and all methods are known to cause pollution. Numerous reports on tons of pollutants caused by alloying [9,10], electroplating [11,12,13] and protective painting [14,15] that cause serious damage to the environment are available. Due to the lack of a better alternative (so far), most commonly, the resistance to the oxidation and electrochemical corrosion of Fe is increased by adding high ratios of Cr and Ni [5,6,16,17,18] which are expensive, and which also have a negative impact on the plasticity of ferroalloys [19].

In fact, Al as an alloying element has been reported to have a positive effect on the oxidation resistance and corrosion resistance of the metallic alloys including Fe [20,21,22,23,24,25,26,27]. In comparison with Cr and Ni, Al is cheap and widely available, which makes it an interesting candidate for industrial use. However, the amount of Al that can be added to Fe is limited. Contents higher than 20 wt.% seriously reduce the plasticity of ferroalloys [19]. In recent years, to address this issue, a low alloying technique combined with a preheating treatment in reducing the atmosphere has been developed for Cu-Al alloys with the additions of <3 mass% Al [20,28,29]. The increase of the oxidation resistance was attributed to the formation of a surface Al_2_O_3_ protective layer that prevents the outward diffusion of Cu as well as the inward diffusion of O. At the same time, the matrix is covered by an Al_2_O_3_ protective layer, and the matrix does not directly contact air or an aqueous solution, which greatly improves the corrosion resistance of the Cu-based alloy.

In this work, the low alloying technique combined with the preheating treatment is applied to Fe to investigate its effect on the corrosion resistance of ferroalloys. It is expected that an Al_2_O_3_ protective layer can be formed on the surface of preheated Fe-Al alloys to improve its corrosion resistance by preventing the outward diffusion of Fe and inward diffusion of O. The low Fe-Al alloy is prepared by adding 1.0 mass% Al into Fe, and the alloy is preheated in a high purity H_2_ atmosphere and subsequently oxidized in a pure oxygen atmosphere. The resistance of the preheated Fe-Al alloy to oxidation at 673 K in pure O_2_ and to electrochemical corrosion in 5 wt.% NaCl solution will be measured. Correspondingly, the surface morphology, structure and the composition of the protective layer formed on the preheated Fe-Al alloy will also be characterized.

## 2. Materials and Experimental Procedures

Fe-Al alloys with 1.0 mass% Al are made from pure Fe (99.999 mass%) and Al (99.9999 mass%) by repeated melting in a vacuum electric arc furnace (DHL-300, SKY Technology Development CO., Shenyang, China) with a nonconsumable electrode under a protective atmosphere (99.999% Ar). The composition is chosen below the solubility limit of Al in Fe [30]. That is, there is no intermetallic compound in the sample to ensure its processability. The sample ingots were cut and cold rolled into thin plates with a thickness of 0.5 mm, and then subjected to mechanical and electrolytic polishing, where the electrolytic polishing current is 0.5 A and the reaction time is 1 min. After that, the samples were preheated in a high purity H_2_ atmosphere (99.9999%) at 101,325 Pa and 1073 K for 1440 min, referred to as P-FeAl. The samples used for the oxidation experiment were stamped into a round piece with a diameter of 4 mm, and the sample used for the electrochemical corrosion experiment was cut into a rectangular piece with a length of 10 mm and a width of 20 mm. During this process, the surface of the sample is covered with high-quality sulfuric acid paper to prevent external pollution and damage to the sample.

The mass gain of the preheated sample and those control samples with pure Fe and unpreheated Fe-Al alloys (uP-FeAl) during oxidation was measured using a thermogravimetric method (METTLER 1100LF, Mettler Toledo, Switzerland). When a sample is heated to the required test temperature in the reaction gas, the increase in mass is recorded. A scanning electron microscope (SEM, JEOL, Tokyo, Japan) and Energy Dispersive X-ray Spectroscopy (EDS, JEOL, Tokyo, Japan) were used to analyze the surface. The detection of surface elements was carried out by X-ray photoelectron spectroscopy (XPS, ESCALAB 250Xi, Thermo Fisher Scientific Inc., Waltham, MA USA). Transmission electron microscopy (TEM, Jeol-NEOARM200F, Jeol, Tokyo, Japan) analysis operating at 200 kV was performed to observe the microstructure of the Al_2_O_3_ layer on the preheated Fe-Al alloy.

The corrosion resistance was assessed with an electrochemical impedance spectroscopy (EIS) and potentiodynamic polarization measurement operated by a CHI660E electrochemical workstation (SUMAT, Beijing, China). The sample was installed in an electrochemical cell and exposed to solutions of 5 wt.% NaCl for 30 min. EIS spectral were then recorded at the open circuit potential (OCP), and measured with a three-electrode configuration, consisting of the sample as the working electrode (surface area 1 cm^2^), a platinum gauze counter electrode and a saturated calomel electrode (SCE) as the reference electrode in a Faraday cage. The EIS experiments were conducted in the frequency range of 10^5^ Hz to 10^−2^ Hz at OCP by applying 10 mV sinusoidal amplitude. Potentiodynamic polarizations were performed after 30 min exposure to solutions of 5 wt.% NaCl. The samples were polarized using a scanning rate of 10 mV/s and a scanning range of − 0.3 V (vs. OCP) ~ 0 V. The solution was open to air and not stirred during the measurement. All experiments were performed at room temperature.

## 3. Results and Discussion

**Figure 1:**
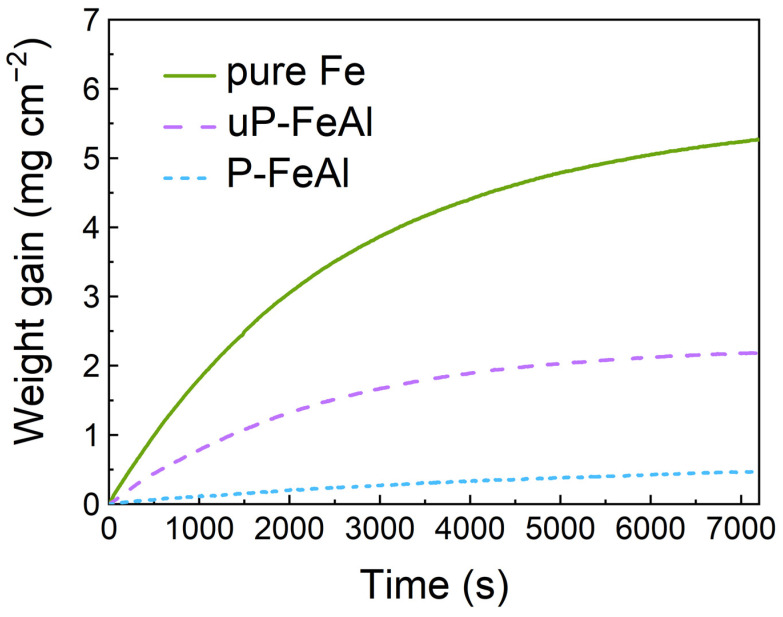
Weight gain curves of pure Fe, uP-FeAl and P-FeAl oxidized at 673 K in 0.1 MPa O_2_ for 120 min showing the superior oxidation resistance of preheated Fe-Al alloys.

To see the effects of the preheating treatment on the oxidation resistance of low Fe-Al alloys, the weight gain curves of pure Fe, uP-FeAl and P-FeAl recorded during oxidation at 673 K in 0.1 MPa O_2_ are given in Figure 1. The weight gain of pure Fe increases the fastest as the oxidation proceeds. In comparison, the weight gain of uP-FeAl is significantly lowered, but still visibly increases, meaning that uP-FeAl is also oxidized but slower than pure Fe. Interestingly, the weight gain of P-FeAl is the lowest and increases the slowest, showing that the oxidation resistance of Fe-Al alloys is significantly improved after the preheating treatment.

**Figure 2:**
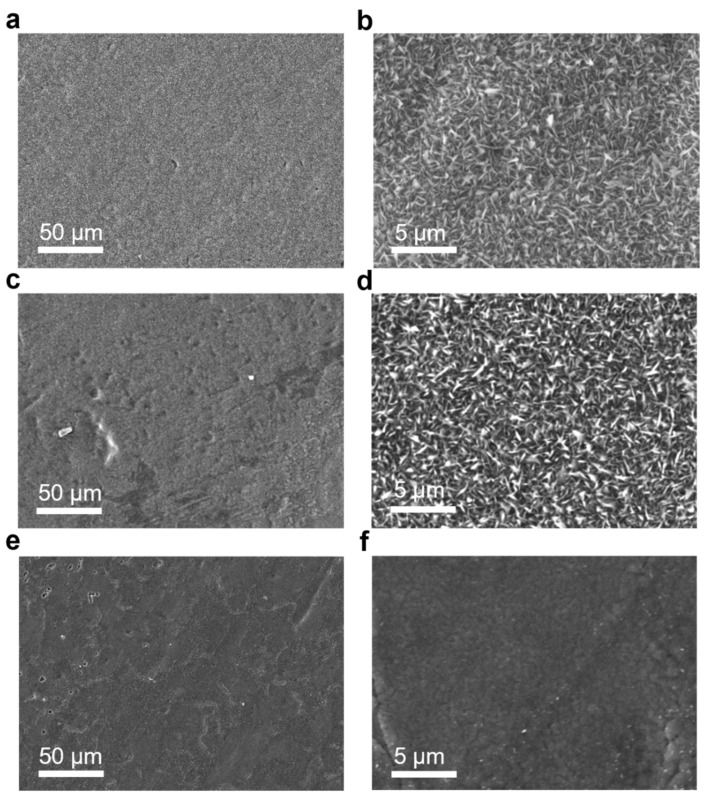
SEM images of surface (oxide) morphologies of pure Fe, uP-FeAl and P-FeAl after oxidation in hydrogen with residual oxygen content of 0.1 MPa at 673 K for 2880 min. (**a**,**b**) pure Fe and (**c**,**d**) uP-FeAl with oxide flakes and whiskers; (**e**,**f**) P-FeAl covered by a uniform and dense oxide layer.

To further evaluate the oxidation resistance of low Fe-Al alloys after preheating, the SEM images of the surface morphology of pure Fe, uP-FeAl and P-FeAl oxidized at 673 K for 2880 min in 0.1 MPa O_2_ are shown in Figure 2. The micrographs of oxidized pure Fe in Figure 2a,b and uP-FeAl in Figure 2c,d show significantly rough surfaces in comparison with that of P-FeAl. Oxide whiskers grown during the oxidation fully cover the alloy surface of pure Fe and uP-FeAl. The whiskers are composed of Fe_2_O_3_ [17]. The surface of P-FeAl in Figure 2e,f is strikingly smooth without oxide grains or whiskers. The oxide morphology images support the previous results of the superior antioxidant ability of P-FeAl from the weight gain curve in Figure 1.

**Figure 3:**
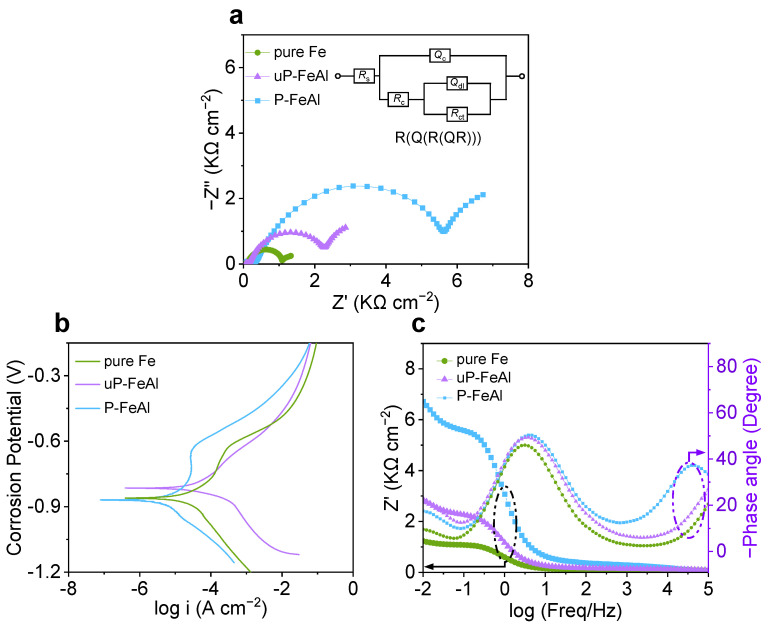
Electrochemical corrosion curves of pure Fe, uP-FeAl and P-FeAl tested in 5 wt.% NaCl solution, with (**a**) EIS diagram at E_corr_, (**b**) Potentiodynamic polarization curves and (**c**) Bode and phase angle plots. Inset of (**a**) shows equivalent circuit model for EIS in (**a**) with R_s_: solution resistance, R_c_: film resistance, Q_c_: film capacitance, Q_dl_: double layer capacitance, R_ct_: charge transfer resistance.

To evaluate the corrosion resistance of one metal or alloy, one efficient way is to provide its electrochemical corrosion diagram, where a high polarization impedance and low corrosion current mean excellent corrosion resistance [31,32,33,34,35,36,37]. In this work, the electrochemical corrosion diagram of pure Fe, uP-FeAl and P-FeAl measured in 5 wt.% NaCl solution is given in Figure 3, with the electrochemical impedance spectroscopy (EIS) diagram in (a), the potentiodynamic polarization curves in (b), and the bode and phase angle plots in (c). In Figure 3a, the EIS curve of P-FeAl has the largest diameter of the semicircle, indicating that it has the highest resistance among the three. With reference to previous studies [38], a two time-constant equivalent circuit model inserted in Figure 3a is constructed to illustrate the electrochemical impedance of a sample that is exposed to the solution of 5 wt.% NaCl. In this model, *R*_s_ represents the solution resistance between the reference electrode and working electrode, the first time-constant represents the resistance of the Al_2_O_3_ protective layer (R_c_) and its capacitance (Q_c_), while the second one describes the electrochemical processes (corrosion) at the substrate in terms of the charge transfer resistance (R_ct_) and the double layer capacitance (Q_dl_). Using such an equivalent circuit model, the fitting result gives that the charge transfer resistance of P-FeAl (R_ct_ = 4948 Ω) is significantly higher than those of pure Fe (R_ct_ = 788 Ω) and uP-FeAl (R_ct_ = 1906 Ω). In Figure 3b, the potentiodynamic polarization curve can be adopted to obtain the corrosion current by the extrapolation method. Due to this, one sees that the corrosion potential (E_corr_) of P-FeAl (E_corr_ = −0.87 V) is lower than that of uP-FeAl (E_corr_ = −0.81 V) and pure Fe (E_corr_ = −0.86 V), and the corrosion current of P-FeAl (I_P-FeAl_ = 9.8 µA cm^−2^) is significantly lower than those of pure Fe (I_pure Fe_ = 35.9 µA cm^−2^) and uP-FeAl (I_uP-FeAl_ = 79.6 µA cm^−2^). Figure 3c shows the Bode and phase angle plots of P-FeAl, pure Fe and uP-FeAl. The phase angle plots show two time-constants; one is for the Al_2_O_3_ protective layer (high frequency range 10^3^–10^5^ Hz), the other is for the electrochemical activity at the matrix (middle frequency range 10^−1^–10^2^ Hz). As for the Bode plot, it can provide the polarization resistance (R_p_) from the difference in the real impedance at a lower and higher frequency [34]. In light of this, P-FeAl has the highest impedance modulus value after 30 min exposure to the 5 wt.% NaCl solution at a low frequency region. Interestingly, the R_p_ of P-FeAl (R_p_ = 6656 Ω) is considerably higher than those of pure Fe (R_p_ = 1157 Ω) and uP-FeAl (R_p_ = 2786 Ω). All these results show that P-FeAl possesses a high corrosion resistance.

In Figure 1, Figure 2 and Figure 3, the excellent corrosion resistance of P-FeAl alloys should be attributed to the preheating treatment prior to the oxidation or corrosion experiments, and a surface protective layer that be formed might be responsible for the improvement. This will be further investigated in the following.

**Figure 4:**
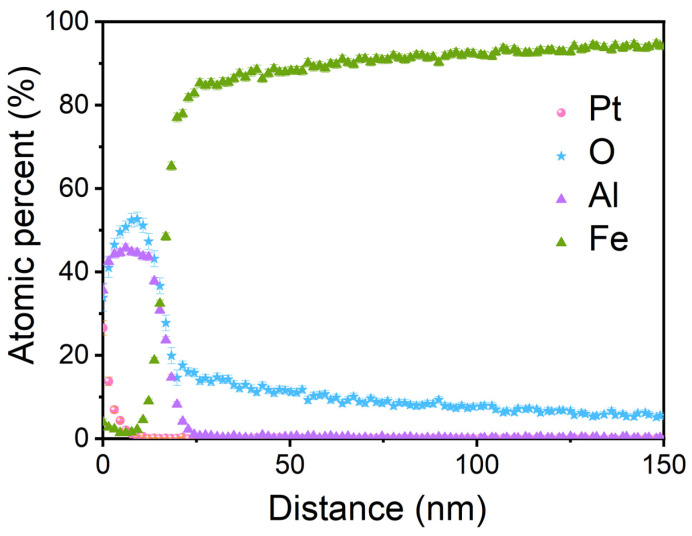
EDS concentration depth profiles of Fe, Al and O measured on P-FeAl.

Figure 4 shows the EDS concentration depth profiles of Fe, Al and O measured on P-FeAl. In the vicinity of the surface, the signals of Al and O are strong, but that of Fe is negligibly weak. As the distance increases, the signals of Al and O decline deeply to a low level, especially for Al, while that of Fe increases quickly up to the maximum level. The depth profiles suggest the accumulation of Al and O in the vicinity of the surface, showing that a thin protective Al_2_O_3_ layer about 20 nm thick is formed on the surface of FeAl during the preheating treatment. The aluminum oxide layer formation is originated from the outward segregation of Al to the surface vicinity. Note that the composition of the aluminum oxide layer is not exactly Al_2_O_3_ but higher in Al content. In addition to this, the profiles show further that oxygen is solved in the Fe-Al alloy, but the level is not in accordance with any stochiometric iron oxide.

**Figure 5:**
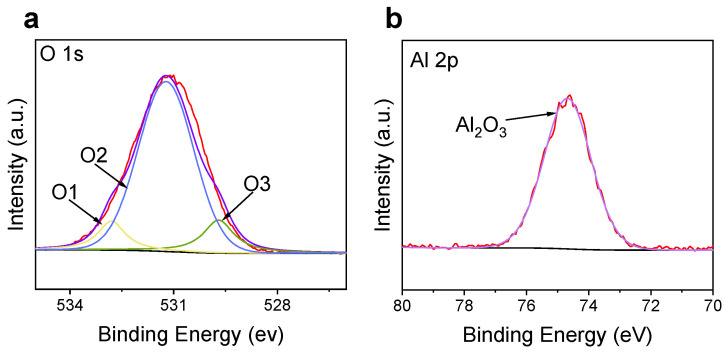
The XPS pattern of the P-FeAl surface confirm the formation of Al_2_O_3_ oxide layer with the characteristic peak positions for (**a**) O 1s and (**b**) Al 2p.

To characterize the composition of the surface protective layer, Figure 5 gives the XPS pattern of the P-FeAl surface. The O 1s spectrum displayed three characteristic peaks of metal–oxygen bonds (529.7 eV for O1), defect sites with a low oxygen coordination (531.2 eV for O2), and hydroxyl groups (532.8 eV for O3), consistent with that reported in the literature [39]. A further characteristic peak appears near 74.3 eV, which comes from Al 2p, also consistent with those reported results [39,40]. These measurements suggest that an Al_2_O_3_ is formed on the surface of P-FeAl, which should be responsible for the improvement in the oxidation resistance and corrosion resistance of P-FeAl.

**Figure 6:**
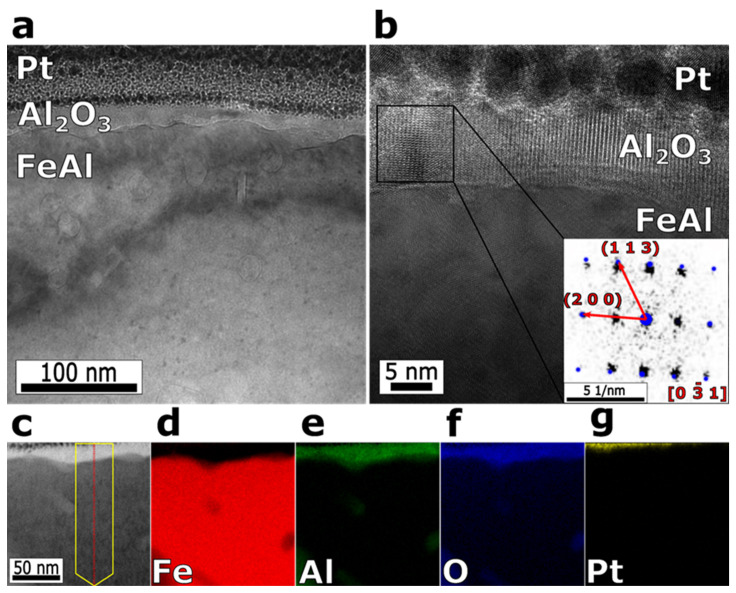
(**a**) BF TEM image of P-FeAl cross section. (**b**) BF TEM image of P-FeAl cross section, where the inset gives FFT image Al_2_O_3_ phase with overlay fit of single crystal^®^. Fitted orientation $\left[0\;\bar{3}\;1\right]$ of orthorhombic Al_2_O_3_ (blue). (**c**) BF TEM image of P-FeAl cross section with corresponding EDS image of (**d**) Fe, (**e**) Al, (**f**) O and (**g**) Pt.

To observe the microstructure of the surface protective layer, a TEM cross section of P-FeAl with EDS mapping of Al, O and Fe are given in Figure 6. In the brightfield image of the surface region shown in Figure 6a, a dense aluminum oxide layer formed on the surface is visible. The mean thickness is 20 ± 5 nm. Figure 6b shows an enlarged view of (a), where the Al_2_O_3_ protective layer can be clearly observed. The Fast Fourier Transform (FFT) inset of the crystalline aluminum oxide layer in Figure 6b was indexed, using the singlecrystal^®^ 4 software, as Al_2_O_3_ orthorhombic (SG 33) structure in $\left[0\;\bar{3}\;1\right]$ orientation. Undoubtedly, this Al_2_O_3_ surface layer is the key to the improvement of the corrosion resistance of P-FeAl. Figure 6d–g exhibit the EDS mapping of Fe, Al, O and Pt over the cross section of P-FeAl as (c). The accumulation of Al and O can be found in the vicinity of the surface of P-FeAl, corresponding to Figure 6. The elemental distributions confirm the formation of a thin protective Al_2_O_3_ layer on the surface of P-FeAl during the preheating treatment. It should be noted here that, in Figure 6d–g, the signals of Al and O can also be observed at the interior place of the Fe-Al base a little further away from the surface, suggesting the internal oxidation of Al in Fe during the preheating process. The internal oxidation is attributed to the solution of O in Fe as shown in Figure 4. Unfortunately, this will lead to a decrease in the actual concentration of Al in Fe, limiting further growth of the surface Al_2_O_3_ layer.

## 4. Conclusions

In summary, P-FeAl is prepared with dilute Fe-Al alloys (1.0 mass% Al) as a precursor through a preheating treatment in an H_2_ atmosphere. After the preheating treatment, P-FeAl shows excellent oxidation resistance. Compared to pure Fe and uP-FeAl, the mass gain of P-FeAl during the oxidation in 0.1 MPa O_2_ at 673 K for 2880 min is much lower, where almost no oxides can be observed on its surface. In the electrochemical corrosion test, P-FeAl also showed excellent electrochemical corrosion resistance with R_p_ = 6656 Ω and I_P-FeAl_ = 9.8 µA cm^−2^, which is significantly better than pure Fe (R_p_ = 1157 Ω and I_pure Fe_ = 35.9 µA cm^−2^) and uP-FeAl (R_p_ = 2786 Ω and I_uP-FeAl_ = 79.6 µA cm^−2^). These improvements are attributed to an Al_2_O_3_ protective layer about 20 nm thick, which is self-formed on the surface due to the reaction of Al outward diffusion from the inner part of the FeAl base with an O_2_ remnant in the annealing atmosphere during the preheating treatment. Such a thin protective Al_2_O_3_ layer can prevent the diffusion of atoms or ions through it during the corrosion process.

## Data Availability

Not applicable.
